# A case of multicentric pancreatic mixed acinar-ductal carcinoma diagnosed by a yogurt-like cell clump flowing from the papilla of Vater

**DOI:** 10.1186/s12876-017-0575-z

**Published:** 2017-01-23

**Authors:** Yoshihiro Kishida, Hiroyuki Matsubayashi, Keiko Sasaki, Shinsaku Honda, Sunao Uemura, Katsuhiko Uesaka, Akiko Todaka, Hiroyuki Ono

**Affiliations:** 1 Division of Endoscopy, Shizuoka Cancer Center, 1007 Shimonagakubo, Nagaizumi, Suntogun, Shizuoka, 411-8777 Japan; 2 Division of Pathology, Shizuoka Cancer Center, Shizuoka, Japan; 3 Division of Hepato-Pancreato-Biliary Surgery, Shizuoka Cancer Center, Shizuoka, Japan; 4 Division of Gastrointestinal Oncology, Shizuoka Cancer Center, Shizuoka, Japan

**Keywords:** Pancreas, Mixed, Acinar cell carcinoma, Ductal adenocarcinoma, Endoscopic retrograde pancreatography, Biopsy, Case report

## Abstract

**Background:**

Histological confirmation is needed when the pancreatic lesions is uncertain for neoplastic or nonneoplastic. Current case with multicentric pancreatic carcinomas showing indefinite clinical images was successfully diagnosed by a biopsy of a novel object expelled from the papilla.

**Case presentation:**

A 71-year-old male was referred because of elevated serum pancreatic enzymes. Computed tomography revealed an unclear low-density area in the pancreatic body without evidence of tumor and mild dilation of the upstream main pancreatic duct (MPD). Other images, including abdominal ultrasound, endoscopic ultrasound, and magnetic resonance imaging, did not demonstrate cancerous findings. Endoscopic retrograde cholangiopancreatography showed a crab-claw-like obstruction in the MPD. Surprisingly, the component constituting the obstruction was moved by contrast injection and spilled out of the papilla orifice as a yogurt-like white object. Biopsy of this object by histology revealed a cancer cell clump. Pancreatectomy was performed, and pathology of the resected pancreas showed multiple nodular tumors replacing the acini and extending into the MPD. These neoplasms histologically resembled mixed acinar-ductal carcinoma.

**Conclusion:**

Current report presented a rare tumor with multicentric pancreatic lesions, preoperatively diagnosed by a biopsy of an uncommon substance.

## Background

In the diagnosis of solid pancreatic tumors, non-invasive image examinations, including ultrasonography (US), endoscopic ultrasonography (EUS), computed tomography (CT), and magnetic resonance imaging (MRI), are effective [1–3], but they have limitations for definitive diagnosis [2]. EUS-guided fine needle aspiration (EUS-FNA) is an excellent tool to obtain histological materials, but it is effective only when the target is visible by EUS [4]. Endoscopic retrograde cholangiopancreatography (ERCP) is also a useful diagnostic tool that enables procurement of pathological samples by aspirated or brush cytology and biopsy [5]. These histological samples are usually pancreatic juice, mucin, brushing materials, lavage fluid, and biopsied pancreatic ductal tissues.

We present a rare case of pancreatic poorly differentiated carcinoma that could not be diagnosed by imaging examinations but could be diagnosed by a yogurt-like white substance that spilled out from the papilla of Vater during endoscopic retrograde pancreatography (ERP). The substance demonstrated unique histologic features.

## Case presentation

**Fig. 1:**
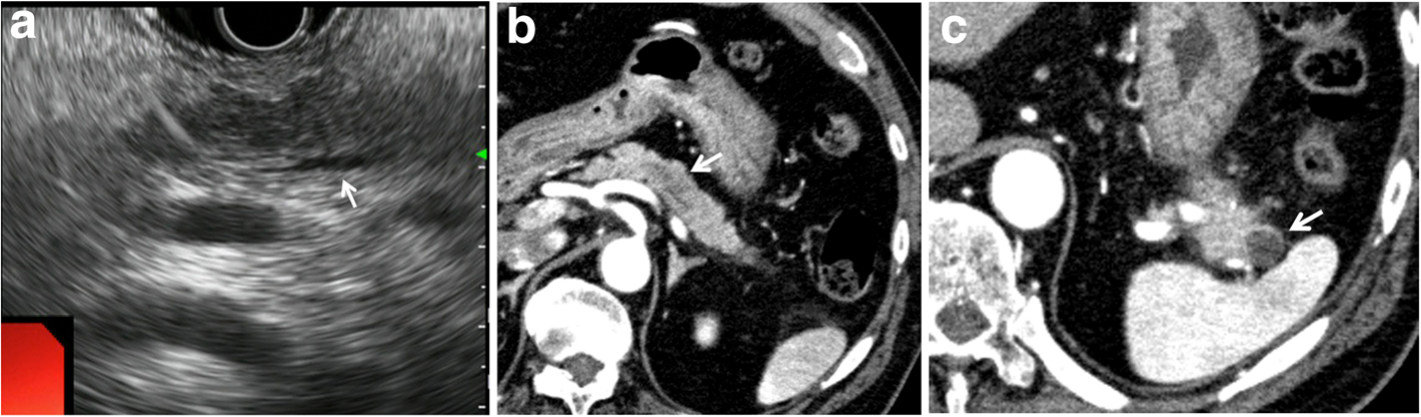
**a** Endoscopic ultrasonography showed a diffuse low-echoic area at the pancreatic body (Pb) and a slightly dilated upstream main pancreatic duct (MPD) (white arrow). **b** Enhanced computed tomography (CT) demonstrated an ill-defined, low-density area at the Pb (white arrow). **c** In the pancreatic tale (Pt), CT revealed a well-demarcated, low-density lesion with faint marginal enhancement

The patient was a 71-year-old male who was referred for an elevated level of serum amylase. He had no symptoms and no previous history or family history. He used to smoke 20-cigarette a day for 40 years but stopped it 10 years before, and drank alcohol occasionally. Laboratory tests demonstrated elevated serum amylase (234 U/L, normal: 37–125 U/L), pancreatic amylase (193 U/L, normal: 21–64 U/L), and cancer antigen (CA) 19–9 (38 U/mL, normal: <37 U/mL). Abdominal US and EUS showed a diffuse low-echoic area around the pancreatic body (Pb) and a mildly enlarged main pancreatic duct (MPD) upstream. This lesion was ill-defined and looked like chronic pancreatitis (Fig. 1a). Enhanced CT revealed an unclear low-density area in the Pb (Fig. 1b). MRI also demonstrated a poorly defined area of low-intensity signal in T1-weighted imaging (T1W) and of iso-intensity in T2-weighted imaging (T2W). Diffusion weighted imaging (DWI) showed an attenuated diffusion level around the Pb, and magnetic resonance cholangiopancreatography (MRCP) showed a segmental stricture of the MPD at the Pb with mild dilation upstream. The second lesion, visible at the pancreas tail (Pt), was a well-demarcated, low-density lesion with faint enhancement at its margin by CT (Fig. 1c). This lesion was visualized as low-intensity signal in T1W, as high-intensity signal in T2W, and as high-intensity signal in DWI, but was invisible in MRCP and enhancement was poor in contrast-enhanced MRI. ^18^F-fluorodeoxyglucose-positron emission tomography (FDG-PET) revealed no abnormal uptake in these lesions. From these findings, chronic pancreatitis was suspected for the lesion at the Pb and a pancreatic cyst for the lesion at the Pt.

**Fig. 2:**
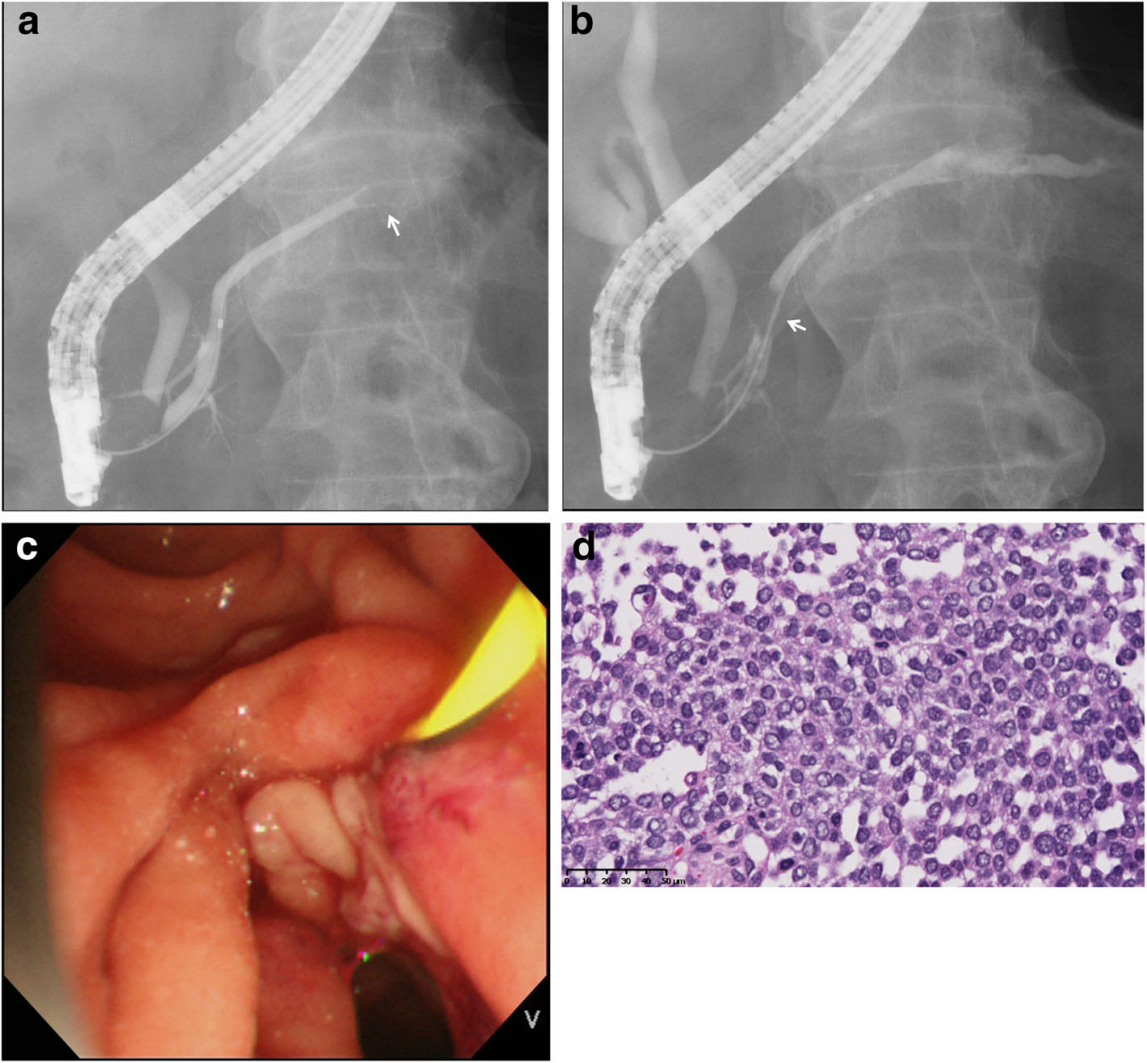
**a** Endoscopic retrograde pancreatography revealed a crab-craw-like obstruction in the MPD (white arrow). **b** Contrast injection caused the occluding object to move downstream. **c** A yogurt-like white substance spilled from the major papilla orifice. **d** Histology of a biopsy sample showed poorly differentiated carcinoma (HE, x40)

Endoscopic retrograde pancreatography demonstrated a crab-claw-like obstruction in the MPD (Fig. 2a). However, when the contrast was injected in the upstream duct, the substance comprising the obstruction gradually moved toward the pancreas head (Fig. 2b). The substance flowed from the papilla orifice as a yogurt-like white object (Fig. 2c). Biopsy of the white object showed clumps of poorly differentiated carcinoma cells (Fig. 2d). Our preoperative diagnosis was carcinoma of the Pb associated with a suspicious of upstream cyst, and distal pancreatectomy was performed.

**Fig. 3:**
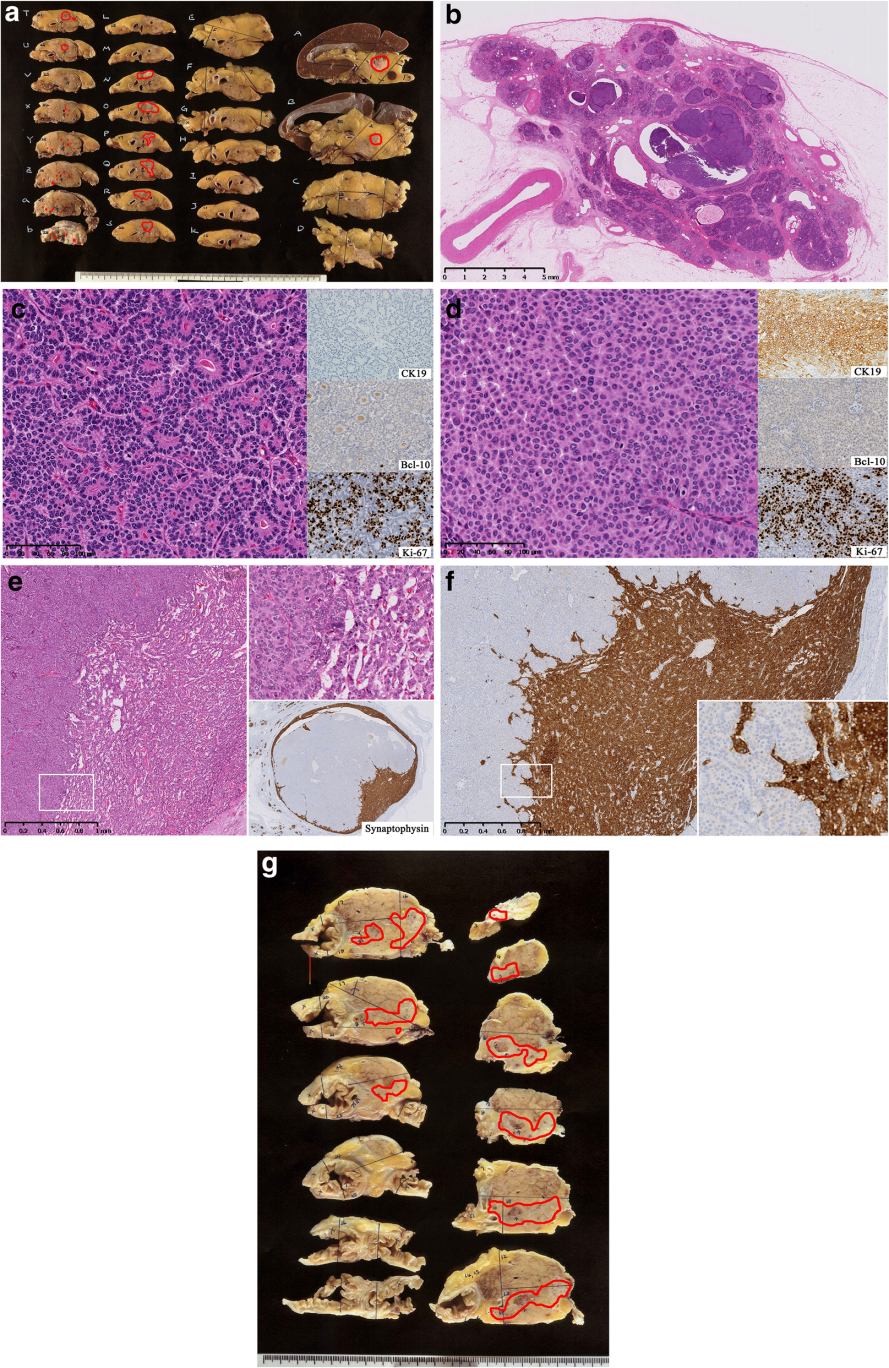
**a** Macroscopic view of the cut surface of resected pancreas body showing the tumor lesions marked in red circles. **b** Loupe imaging of the pancreatic body demonstrated the tumor replacing acinar lobules and occupying the MPD. **c**, **d**﻿﻿ The tumor cells showed basally aligned nuclei and eosinophilic cytoplasm and formed an acinar structure **c** and a solid structure **d**. **e** A tumor of the pancreatic tail displayed a well-demarcated solid mass, included in a component of neuroendocrine tumor. **f** Gross image of additionally resected pancreas head also showed multiple neoplastic lesions, circled in red

In the macroscopic view of the thin-sliced resected specimen, two independent lesions were recognized in the Pb and Pt (Fig. 3a). The loupe image of the hematoxylin and eosin (HE) section revealed multiple clusters of eosinophilic neoplasms replacing the pancreas acini and extending into the MPD (Fig. 3b). Histologically, the tumor formed an acinar or sheet-like structure, composed of cells with eosinophilic cytoplasm and basally aligned, oval-shaped nuclei (Fig. 3c) and solid structure without any acinar or ductal structure in some area (Fig. 3d), and was compatible with the tissue collected during the preoperative ERP. Ki-67 was positive in the both areas (Fig. 3c, d). These histological findings resembled those of acinar cell carcinoma (ACC), but trypsin, lipase, and Bcl-10 were all negative by immunohistochemistry (Fig. 3c); the source of the antibodies for immunohistochemistry staining is shown in Table 1. Instead, cytokeratin 19 (CK19), cytokeratin 7 (CK7), and MUC 1 were slightly positive especially in the solid area, which indicated differentiation of ductal adenocarcinoma (Fig. 3d). The tumor grew replacing the surrounding normal less-atrophic pancreatic body tissue, but interstitium was almost kept normal, and the findings of pushing-border or infiltration were relatively poor. The maximal size of this tumor was 35 mm.

**Table 1:** Immunohistochemical methods

Antibody	Company	Titration
Bcl-10	Santa Cruz	1:2,000
Trypsin	Abcam	1:200
Lipase	Abcam	1:20,000
Cytokeratin 7	DAKO	1:800
Cytokeratin 19	PROGEN Biotechnik	1:800
CEA	Leika Biosystems	1:200
CA19-9	DAKO	1:50
MUC1	Novo Castra	1:100
Chromogranin A	DAKO	1:5,000
Synaptophysin	Leica Biosystems	1:400
Ki-67	DAKO	1:100

Histology of another solitary tumor at the Pt was mostly the same as that of the tumor in the Pb (Fig. 3a). It was contained in a component of neuroendocrine tumor arranged in a ribbon-like pattern, with diffuse expression of synaptophysin and chromogranin A, and negative for Ki-67 (<2%) (Figs. 3e). Thus it was considered that the tumor metastasized in the neuroendocrine tumor in the Pt. The size of this tumor was 17 mm.

In addition to the above two lesions, multiple neoplastic cells nests, replacing the acini and creeping into the small duct, were microscopically detected in the proximal section of the specimen (Fig. 3a). Immunostaining of CK19 was positive within the neoplastic cells and progressed through the excretory ducts. These foci were not evident in the preoperative images, by rapid pathological diagnosis, or even in the macroscopic view of the sliced pancreatic specimens.

The overall pathological diagnosis by UICC criteria was mixed acinar-ductal carcinoma, pT2N0M0 pStage IB, with partial endocrine differentiation. Lymphatic permeation was positive, but no venous invasion or lymph nodal metastasis (0/17) were recognized within the resected materials. Neoplastic cells were floating in the pancreatic duct at the cut end, but the cells were not connective with the basal membrane. The patient received adjuvant chemotherapy with S-1 (120 mg/day) for 6 months and was followed up because of his advanced age.

Twenty-two months after the operation, recurrence occurred in the remaining pancreas. Again, excretion of the yogurt-like substance was recognized at the major papilla. Additional pancreaticoduodenectomy was performed, and the resected specimen showed the same but more poorly differentiated neoplasm spreading into the MPD and branch pancreatic ducts, 45 mm in size (Fig. 3f). A small nodule of hepatic metastasis was also found and removed during the operation. The patient again received S-1 as adjuvant chemotherapy, and is still alive until 13 months after the additional surgery with progression of liver metastasis.

## Discussion

We report here a case of multiple mixed acinar-ductal carcinoma of the pancreas demonstrating mostly atypical images and partially invisible by imaging, which was difficult to diagnose without obtaining histological evidence. The crucial pathological sample was a yogurt-like cell clump that flowed out of the papilla during ERP. This unique phenomenon helped us diagnose and treat this rare pancreatic tumor. To the best of our knowledge, such an occurrence has not yet been reported.

Histological samples from pancreatic cancer, obtained by EUS-FNA, forceps biopsy, aspiration, and/or brushing cytology, are usually solid tissue, liquid, or sometimes mucinous. The current sample, a “yogurt-like” or”gel-like” whitish substance, was distinctive. Presumably, the white color was due to its high cellularity, and the viscosity reflected the intercellular adhesion (Figs. 2d, 3b). Histology of the current tumor resembled pancreatic ACC, which characteristically shows a sheet-like spreading of neoplastic cells without dense interstitial tissues and sometimes accompanies partial differentiation to ductal dilatation (about 30%) [6, 7], neuroendocrine neoplasm (14–27%) [8–12], and intraductal extension (about 10%) [6]. ACC with pancreatic ductal ingrowth typically shows a nodular or papillary progression pattern, similar to that of intraductal papillary mucinous neoplasm [13], as seen in the current case. This rare but characteristic histology of the MPD may have been reflected in the yogurt-like substance expelled during ERP.

The current case pathologically demonstrated an ACC-like structure by HE staining, but it was negative for trypsin, Bcl-10, and lipase. The sensitivity of this type of immunostaining for ACC is reported to be fairly high: 95–100% for trypsin [12, 14, 15], 82–100% for Bcl-10 [12, 16, 17], and 26–45% for lipase [12, 14, 15]. Our literature survey revealed no cases of ACC showing null expression of Bcl-10, lipase, and trypsin. On the other hand, CK7, CK19, CEA, CA19-9, and MUC1 are highly sensitive for ordinary pancreatic ductal adenocarcinoma (sensitivity: 96%, 100%, 85%, 75%, and 88%, respectively) [10, 18–21], and CK7, CK19, and MUC1 were partially positive in the current tumor. These findings suggested that although HE section indicated ACC, the phenotype of the ACC were vanishing and phenotype of ductal adenocarcinoma appeared in some severe atypicus area. Hence, we pathologically classified the current tumor as mixed acinar-ductal carcinoma.

Although the histology of ACC-like component was quite typical for ACC, excepting the immunohistochemistry, imaging of the current tumor was not typical, as ACC usually forms a huge and exophytic, well-circumscribed, hypovascular mass [22–26]. Toll et al. report a similar case of ACC showing multifocal intraductal extensions and replacements of normal pancreatic parenchyma throughout the entire pancreas [27]. The current case demonstrated the same progression pattern, and it was considered as a reason for the poor tumor definition in the preoperative images.

## Conclusion

In summary, we report a rare case of multifocal mixed acinar-ductal carcinoma of the pancreas. The carcinoma showed atypical imaging findings and was diagnosed by pathological examination of a yogurt-like cell clump that flowed out of the papilla during ERP.

## Abbreviations

ACC: Acinar cell carcinoma
CT: Computed tomography
DWI: Diffusion weighted image
ERCP: Endoscopic retrograde cholangiopancreatography
ERP: Endoscopic retrograde pancreatography
EUS: Endoscopic ultrasonography
EUS-FNA: EUS-guided fine needle aspiration
FDG-PET: ^18^F-Fluorodeoxyglucose-positron emission tomography
HE: Hematoxylin and eosin
MPD: Main pancreatic duct
MRI: Magnetic resonance imaging
Pb: Pancreatic body
Pt: Pancreatic tail
T1W: T1-weighted imaging
T2W: T2-weighted imaging
US: Ultrasonography
